# Physics of microbial taxis and behaviours in response to various physical stimuli

**DOI:** 10.1098/rsta.2024.0264

**Published:** 2025-09-11

**Authors:** Takuji Ishikawa, Katsuhiko Sato, Toshihiro Omori, Kenjiro Yoshimura

**Affiliations:** ^1^ Department of Biomedical Engineering, Tohoku University, Sendai, Miyagi, Japan; ^2^ Program of Mathematics and Informatics, Faculty of Science, University of Toyama, Toyama, Japan; ^3^ Department of Finemechanics, Tohoku University, Sendai, Miyagi, Japan; ^4^ Department of Bioscience and Engineering, Shibaura Institute of Technology, Saitama, Japan

**Keywords:** microorganism, gravitaxis, phototaxis, rheotaxis, gyrotaxis, thigmotaxis

## Abstract

The physical environment exerts a profound influence on microbial life. The directional movement of cells in response to their physical environment is understood as taxis, which has been studied in biology as chemotaxis, phototaxis, gravitaxis and so forth. These taxis are induced by physiological, physical or both factors. Nevertheless, the physical picture of cellular responses to the environment at the individual cell scale remains unclear. In this review paper, we therefore aim to provide an overview of the recent progress in the physical depiction of microbial taxis and their behaviour in various environments. Four types of responses are discussed: responses to (i) gravity, (ii) light, (iii) surface and (iv) flow. For each response, a simple physical picture is presented that describes the mechanism of behaviours. Knowledge of physical mechanisms will help us to understand the behaviour of cells in various environments at both the individual and population levels. Finally, the future challenges and prospects in this research field are discussed.

This article is part of the theme issue ‘Biological fluid dynamics: emerging directions’.

## 1. Introduction

Microorganisms live in a world that is replete of physical and chemical stimuli. Microorganisms are able to sense and respond to these stimuli, which can be observed in behaviours such as swimming towards (or away from) the stimuli. Some marine microalgae, for example, engage in diurnal vertical movement [1,2]. During the day, they remain in proximity to the water’s surface where light is strong, and at night they sink to deeper water in search of nutrients. Both physics and physiology are involved in the process of diurnal vertical movement. Its extent depends on the species of microorganism. Furthermore, zooplankton also exhibit diurnal vertical movement in response to the movement of prey and predators [3]. The occurrence of a red tide may be attributed to the influence of hydrodynamic stimuli, such as tidal currents, on the diurnal vertical movements of the red tide microalgae. Durham *et al*. [4] demonstrated that the vertical migration of phytoplankton resulted in the formation of layers when the migration was disrupted by shear flow. This example illustrates that the biological function of a red tide is the result of microorganisms responding to physical stimuli, including light, gravity and the flow field. It is crucial to comprehend how microorganisms respond to physical and chemical stimuli and modify their behaviour in order to interpret the function and behaviour of microbial populations [5].

A variety of responses of microorganisms to environmental stimuli have been studied. These include light intensity [6,7], chemical concentration field [8,9], gravity field [10] [11], electric field [12], magnetic field [13], flow field [14], temperature gradient [15], salinity gradient [16], viscosity gradient [17], wall boundary [18], free surface [19] and so forth. Accumulation towards and avoidance away from physicochemical stimuli in microorganisms has been recognized as taxis in the field of biology. In a narrower definition of taxis, accumulation is accompanied by changes in the direction of movement with respect to the direction of stimulus origin. The following known taxis have been identified: phototaxis, chemotaxis, gravitaxis, electrotaxis, magnetotaxis, rheotaxis, thigmotaxis, thermotaxis, viscotaxis and gyrotaxis.

**Figure 1.**
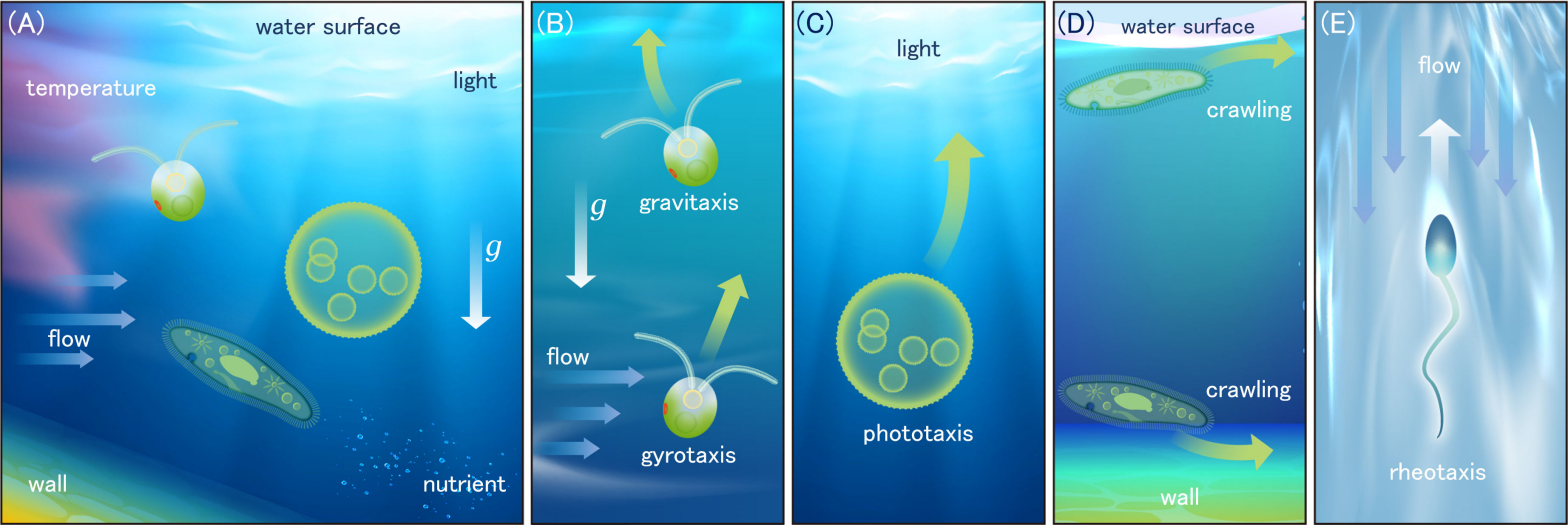
Various responses of microorganisms to environmental stimuli. (A) Microorganisms exposed to various physicochemical stimuli in environments, (B) schematics of gravitaxis and gyrotaxis, (C) schematics of phototaxis, (D) schematics of thigmotaxis and (E) schematics of rheotaxis.

Chemotaxis is a response to chemical substances, and the chemotaxis in *Escherichia coli* has been the subject of long-standing investigation [20]. In the case of *E. coli*, when facing an attractant, the cell swims in a straight line for a longer period with bundled flagella. Conversely, when moving away from the attractant, the frequency of flagellar spreading and tumbling increases. This run-and-tumble mode allows them to accumulate in closer proximity to the attractant. Such chemotaxis can be described physically as a biased random walk [21]. The random walk model is a widely used approach in physics to describe the diffusion of a macroscopic population as individual particles that repeatedly change direction in random directions [22]. By understanding the underlying principles of random walk, it becomes possible to quantitatively describe the biological phenomenon of microbial diffusion as being proportional to the square of the swimming speed and inversely proportional to the frequency of directional changes. This physical picture of random walk has proved invaluable in facilitating a quantitative understanding of microbial chemotaxis. In recent years, the physical mechanisms of chemotaxis as well as other taxis have been rapidly uncovered. This review paper will therefore explain the physical mechanisms of four types of behaviours: gravitaxis, phototaxis, thigmotaxis and rheotaxis, illustrated in figure 1. It will also provide an overview of recent advances in our understanding of these behaviours and the simple mathematical models that describe them.

Gravitaxis or geotaxis is the property of microorganisms, such as microalgae and ciliates, to move in a specific direction in response to a gravitational field [11]. Gravitaxis plays a pivotal role in the diurnal vertical movement of cells and their ascension to the water surface. In the case of a unicellular ciliate *Paramecium*, for example, the property of passive upward swimming is reported to be caused by a combination of anterior–posterior asymmetry in cell shape and the effect of sedimentation due to the higher specific gravity of the cell than the surrounding liquid [23]. Gyrotaxis is the property of microorganisms to swim in a specific direction, determined by a balance between the gravitational and viscous torques. Gyrotaxis thus encompasses both gravitaxis and rheotaxis. Kessler [24,25] demonstrated that the swimming direction of algal cells was directed towards a concentrated beam in tube flow due to gyrotaxis. Section 2 will describe the physics of microbial gravitaxis and gyrotaxis, as well as the recent advances in our understanding of these processes.

Phototaxis is the property of moving in a specific direction in response to light stimuli, which is exhibited by photosynthetic microalgae and other organisms [26]. For instance, a unicellular microalga *Chlamydomonas* is capable of sensing light at its cell organelle, called eyespot, and is able to swim in the direction of light by changing its ciliary beating in response to the stimulus. Recently, a physical model has been developed that can represent the phototactic behaviour of the volvocine algae, such as *Chlamydomonas* and *Volvox*, at the cellular scale [27]. Section 3 will present an overview of recent advances in the physics of microbial phototaxis.

Thigmotaxis is the tendency of an organism to stay close to surfaces. Microorganisms are often found around interfaces, such as water surfaces and wall boundaries. In the case of microbial symbionts, for example, the symbionts undertake complex internal journeys to reach specific cellular compartments or housing organs [28]. The physical environments surrounding the symbionts are geometrically intensely constrained. Microorganisms are capable of modifying their behaviour in response to the geometric constraints, including the ability to escape, crawl or adhere to the interface. Recent experiments with a unicellular ciliate *Tetrahymena* have revealed that crawling at interfaces is mediated by cell shape [29] and ciliary response [18]. In §4, we will explain recent advances in the physics of microbial response to walls and water surfaces.

Rheotaxis is the property of microorganisms to swim against the current. This phenomenon is well-known as fish swimming in rivers, but it is also known that some microorganisms and spermatozoa exhibit rheotaxis. In the case of sperm, rheotaxis plays an important role in sperm guidance over long distances in the mammalian female reproductive tract [30]. The mechanism of rheotaxis is analogous to that of a weathervane facing upwind and can be elucidated by the interplay between the surrounding current and the wall surface [31]. Section 5 will describe an overview of recent advances in the physical understanding of microorganism rheotaxis. In addition, this section covers viscotaxis, which is caused by the viscosity gradient of the surrounding fluid.

As described above, considerable progress has been made in understanding the physical mechanisms underlying microbial responses to gravity, light, interfaces and flow. Nevertheless, numerous unresolved issues remain, including the simultaneous action of multiple physical stimuli, the behaviour of cell populations, and the molecular biological mechanisms of responses. In §6, we will present these outstanding issues and indicate the direction of our future research.

## 2. Responses to gravity

Since the birth of living organisms, gravity has been acting on every living thing on earth, and even microorganisms cannot avoid the effects of gravity. Many microorganisms exhibit a response to gravity, with a tendency to swim in an upward direction (negative gravitaxis). The mechanism of negative gravitaxis has long been investigated in the field of biology [11,32,33], and the mechanism has been explained by physiological, physical or both factors. In this section, we focus on the physical factors and explain the mechanism of gravitaxis caused by bottom-heaviness of cells, asymmetric body shape and adhesion to bubbles. In addition, this section also addresses gyrotaxis, which emerges from the combined effects of gravitaxis and rheotaxis.

When microorganisms, such as a ciliate *Paramecium* and a microalgae *Chlamydomonas*, are cultured, the cells accumulate near the water surface due to negative gravitaxis and descend as a plume. The convection caused by microorganisms is referred to as ‘bioconvection’, a term coined by Platt [34]. Microorganisms that cause bioconvection have greater density than the surrounding fluid, yet tend to swim upwards [35]. When these microorganisms accumulate near the water surface, the upper region of the suspension becomes denser than the lower region, and the fluid becomes unstable. This results in overturning convection analogous to Rayleigh–Bénard convection.

### (a) Gravitaxis due to bottom-heaviness

The Reynolds number is a dimensionless number that describes flow conditions and indicates the ratio of inertial to viscous forces. The half-body length of most microorganisms ranges from about 1 to 100 μm, and they swim at a speed of about 1 to 10 body lengths per second [36]. The Reynolds number, using the density and viscosity of water, ranges from $10^{-5}$ to $10^{-1}$. Therefore, inertia can be neglected and the flow field around a microorganism can be approximated as a Stokes flow. We will discuss the forces acting on a microorganism assuming Stokes flow.

**Figure 2.**
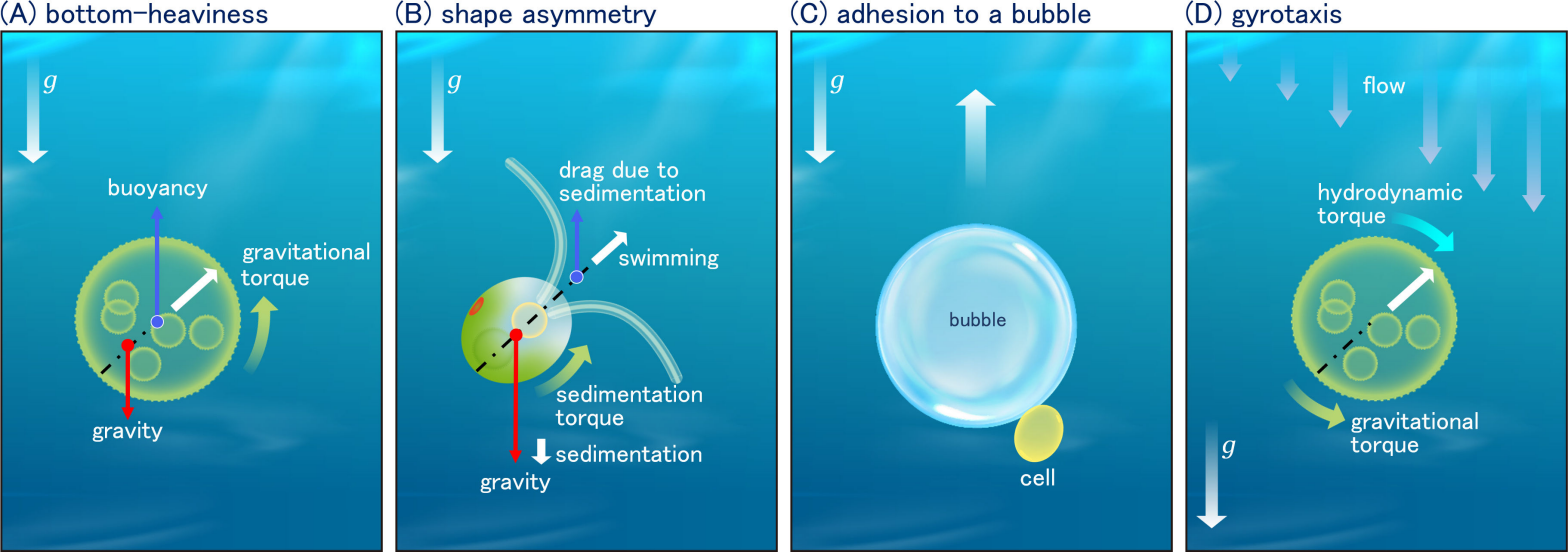
Physical mechanism of gravitaxis and gyrotaxis. (A) Gravitaxis due to bottom-heaviness. The centre of the geometry is indicated by the blue dot, where the buoyancy force is acting. The gravity centre is located slightly posterior to the geometric centre, as indicated by the red dot. As the gravity and buoyancy forces are not aligned vertically, a torque is produced that rotates the body, as indicated by the green arrow. (B) Gravitaxis due to shape asymmetry. In Stokes flow, the sedimentation and swimming dynamics can be considered separately. The centre of drag in sedimentation is indicated by the blue dot. The drag force is not aligned vertically with the gravity force, resulting in a torque that rotates the body vertically upward. (C) Gravitaxis due to bubble adhesion. A microorganism that is denser than the surrounding fluid and has no swimming ability migrates vertically upward by adhering to a bubble it produces. (D) Gyrotaxis is generated by a balance of gravitational (green) and hydrodynamic (blue) torques.

When an object is immersed in water, buoyancy acts on the centre of the geometry, while gravity acts on the centre of gravity. The microalgae *Volvox* is an ideal model for elucidating this phenomenon, as its body shape is nearly spherical and the centre of the geometry is at the centre of the sphere, which is readily identifiable. In figure 2A, the centre of the geometry is indicated by a blue dot, where the buoyancy force is acting. By contrast, the gravity centre of *Volvox* is located slightly posterior to the centre of the sphere, as indicated by a red dot. The posterior of *Volvox* holds a number of dense germ cells, which results in a posterior shift of the gravity centre. When the gravity and buoyancy forces are not aligned vertically, a torque is produced that rotates the body, as indicated by the green arrow in the figure. The bottom heaviness in *Volvox carteri* was measured by Drescher *et al*. [37]. It takes approximately 10−15 s for a *V. carteri* to turn upwards through the bottom-heaviness mechanism. When a small sphere rotates slowly in a liquid, Stokes drag holds between torque T and angular velocity Ω as $T=8\pi \mu R^{3}\Omega$, where μ is the viscosity and R is the radius. Consequently, by measuring the angular velocity of *Volvox*, it is possible to estimate the torque acting on it. Furthermore, the volume of *Volvox* can be determined from its radius, and the buoyancy force can be derived by using the density of the water. The maximum torque acting on *Volvox* is the product of the buoyancy force and the distance between the gravity and geometry centres. Utilizing these relationships, the distance for *V. carteri* can be estimated to be approximately 40 nm. A similar experiment was also conducted for *Chlamydomonas reinhardtii* [38]. The chloroplast of *C. reinhardtii* is cup-shaped and located posterior to the cell body. It is denser than the rest of the body. Their measurement revealed that the distance between the gravity and geometry centres of *C. reinhardtii* is approximately 30 nm.

### (b) Gravitaxis due to shape asymmetry

The mechanism of gravitaxis due to shape asymmetry would be a more challenging phenomenon to comprehend than that due to bottom-heaviness. Flow field around microorganisms can be regarded as Stokes flow with negligible inertia, given that microorganisms are small and swim slowly. In the Stokes flow regime, the flow field is linear and can be superimposed, allowing sedimentation due to gravity and swimming due to ciliary beat to be considered separately and their results can be superimposed [39]. To illustrate this, consider the situation shown in figure 2B, where a microorganism with two cilia, such as *Chlamydomonas*, is swimming diagonally upward. It is assumed that the microorganism is slightly denser than the surrounding fluid and that it sediments at a velocity $V_{s}$ when the cilia stop moving. Conversely, in the absence of gravity, this microorganism is assumed to swim with a velocity $V_{f}$ by ciliary beating. When this microorganism swims under gravity, it swims at a velocity $V_{f}-V_{s}$ due to the combined effects of sedimentation and ciliary beat. This argument implies that the actual velocity can be obtained by superimposing the velocities generated by different forces. We note that in the case of *Paramecium*, the actual swimming velocity is not identical to $V_{f}-V_{s}$, which suggests that other biological responses influence the gravitactic behaviour [40,41].

The discussion now turns to the rotational velocity of the microorganism. In the Stokes flow regime, the rotational velocities resulting from gravity and ciliary beat can also be superimposed. In the absence of gravity, the microorganism is assumed to swim in a straight path diagonally upward. Consequently, no rotational velocity is observed. In the presence of gravity and immobile cilia, on the other hand, the microorganism sediments at a speed $V_{s}$. The gravity centre of the microorganism is located near the centre of the cell body, as indicated by a red dot in figure 2B. An object undergoing sedimentation is subjected to a drag force exerted by the surrounding fluid, with the centre of drag indicated by a blue dot in the figure. The reason why the centre of drag is located anteriorly on the body is because the flow resistance of cilia is considerable. The drag force is directed upward and acts at the centre of drag, which is not aligned vertically with the gravity force. Consequently, a torque is generated that rotates the body vertically upward, as indicated by the green arrow in the figure. Finally, the actual rotational velocity can be obtained by superimposing the rotational velocity due to swimming, which is zero, and the rotational velocity due to sedimentation. This is the mechanism of gravitaxis due to shape asymmetry.

The significance of shape asymmetry in the gravitaxis of *Paramecium* has been reported by Robert *et al.* [23,42]. They concluded that the shape asymmetry is sufficient to account for the observed rotational velocity of *Paramecium*. Conversely, there are reports that biological responses also contribute to the gravitaxis of *Paramecium* [40,41]. Further research is required to substantiate these two claims. Roberts [43] reported the effect of shape asymmetry on the gravitaxis of *Chlamydomonas*. He noted that cilia have a major impact, and again concluded that the shape asymmetry is primarily responsible for upward swimming in *C. reinhardtii*. Kage *et al*. [38] further investigated the gravitaxis of *C. reinhardtii* by comparing the gravitaxis resulting from shape asymmetry and bottom-heaviness. A numerical simulation was performed using a boundary element method, which demonstrated that the rotational velocity generated by shape asymmetry is approximately six times as large as that generated by bottom-heaviness. These findings illustrate the significance of shape asymmetry in gravitaxis.

When *Paramecium* and *Chlamydomonas* cells are suspended in a medium with a specific density greater than that of the cells, the cells migrate downwards and exhibit positive gravitaxis [44,45]. These observations are incompatible with the bottom-heaviness mechanism, as the direction of the torque generated by gravity and buoyancy remains unchanged in the high specific density medium. The result indicates that microorganisms exhibit negative and positive gravitaxis when cells passively sink downward and float upwards, respectively, which is consistent with the shape asymmetry mechanism.

### (c) Gravitaxis due to gas production

Some microorganisms produce gas as intracellular vesicles or extracellular bubbles and move upward due to the buoyancy. This mechanism of vertical migration can also be considered a form of gravitaxis, since gas is actively produced to reduce cell density below that of the surrounding fluid.

There are a number of microorganisms, including cyanobacteria and halobacteria, that have long been known to alter cell density and control floating and sinking by means of gas vesicles in the body [46]. Gas vesicles are hollow intracellular proteinaceous organelles that increase buoyancy and allow negative gravitaxis. An enterobacterium, *Serratia*, for instance, can control the buoyancy force by regulating the gas vesicles and shows environmental adaptation through physiological control of the choice between flagella-based motility and gas vesicle-mediated flotation as alternative taxis modes [47].

Bubble adhesion has been utilized in flotation processes in engineering, such as in the field of wastewater treatment. The efficient floatation of various types of biological cells, such as bacteria, fungi and yeasts, has been studied in dispersed air flotation and dissolved air flotation [48]. However, the phenomenon of cells themselves creating bubbles, attaching to them, and moving to the water surface, as shown in figure 2C, has not been well documented [49]. In the yeast fermentation process, yeast can migrate from the bottom of the container to the water surface by adhering to bubbles produced by itself during the fermentation process. The adhesion of yeast to bubbles is strongly influenced by the hydrophobicity of the yeast cell wall [50]. Srivastava *et al*. [51] investigated the vertical distribution of yeast in two major types of fermentation in breweries, namely top fermentation (ale) and bottom fermentation (lager). They found that for top-fermenting ales, bubble adhesion is the dominant mechanism of vertically upward transport of yeast compared with bubble-induced mixing. By contrast, for bottom-fermenting lagers, bubble-induced mixing dominates over bubble adhesion, although vertically upward transport of yeast is much less than for the top-fermenting ales. The benefits of top-fermenting may include faster growth using oxygen near the top surface and easier nutrient uptake as nutrients are distributed throughout the suspension by the vertical movement of the yeast.

Potentially bubble-producing microorganisms may have a similar ability to adhere to bubbles. Gas-producing bacteria such as *Lactobacillus* and *Clostridium* also produce gases and generate bubbles. It would be interesting to study these species. We note that several animals, such as snails and aquatic insects, can hold air bubbles on their water-repelling bodies in order to reduce their effective density [52,53].

### (d) Gyrotaxis

Gyrotaxis is the directed swimming of microorganisms resulting from a balance of gravitational and hydrodynamic torques. When the background flow has vorticity, as illustrated in figure 2D, the microorganism rotates clockwise due to the hydrodynamic torque. If the microorganism also exhibits gravitaxis, due to bottom-heaviness or shape asymmetry, the gravitational torque is generated in the counter-clockwise direction. Given that the gravitational torque varies with the tilt angle of the microorganism, there may be an angle at which the hydrodynamic and gravitational torques are balanced. The microorganism swims at this balancing angle, resulting in gyrotaxis exhibiting angled migration with respect to the direction of gravity.

Kessler [24] observed *Chlamydomonas* accumulating on a tube axis amid a descending flow. The gravitaxis mechanism generates a torque that directs the cell upwards, as indicated by the green arrow in the figure 2D. On the other hand, the descending flow in the tube causes the cell to rotate towards the tube axis, because the flow is faster at the centre of the tube. When these two torques are balanced at a certain tilt angle, the cell swims towards the tube axis. Gyrotaxis in pipe flow is being actively studied, including the effects of cell motility on diffusivity [54] and the stability of gyrotactic patterns [55]. Gyrotaxis may also play a role in the clustering of microorganisms in turbulent flow. De Lillo *et al*. [56] demonstrated that fluid acceleration generates multifractal clustering of microorganisms due to gyrotaxis. In a recent review, Qiu *et al*. [57] overviewed gyrotaxis of microorganisms in turbulent flows. Further details on the turbulent effect will be presented in §5.

## 3. Responses to light

The ability of microorganisms to move towards or away from light, known as phototaxis, is crucial for the efficient performance of photosynthesis and the avoidance of damage from light [26]. Since numerous excellent review papers have been published on the molecular mechanisms of phototaxis in microorganisms [6,26,58,59], this section will focus on the ‘physical’ mechanisms of phototaxis from a mechanical and mathematical perspective.

The mechanisms of phototaxis in microorganisms can be broadly classified into two categories. The first category encompasses a biased random movement, which is characterized by repeated run-and-tumble motions and change in the frequency of tumbling in response to light intensity, as observed in phototactic bacteria and archaea. This behaviour enables microorganisms to perform phototaxis in a light-intensity gradient [26]. The second mechanism is more deterministic and involves directional movement. This type of taxis is defined as taxis in the narrow sense as mentioned in the Introduction. Microorganisms, including unicellular and multicellular eukaryotes such as *Chlamydomonas* and *Volvox*, swim in open water by using cilia and flagella, drawing a spiral trajectory in three-dimensional space. The light stimuli received by photoreceptors (eyespots) on the body modulate the amplitude and beat frequency of ciliary beating, which leads to a change in the direction of the axis of the helical trajectory. This ultimately results in the achievement of phototaxis in three-dimensional space. The former mechanism, the biased random walk strategy, has an analogy with that of chemotaxis in bacteria [60]. As the mechanism has been well described in numerous papers, this section will focus on the latter deterministic mechanism.

### (a) Mathematical description of three-dimensional movement

**Figure 3.**
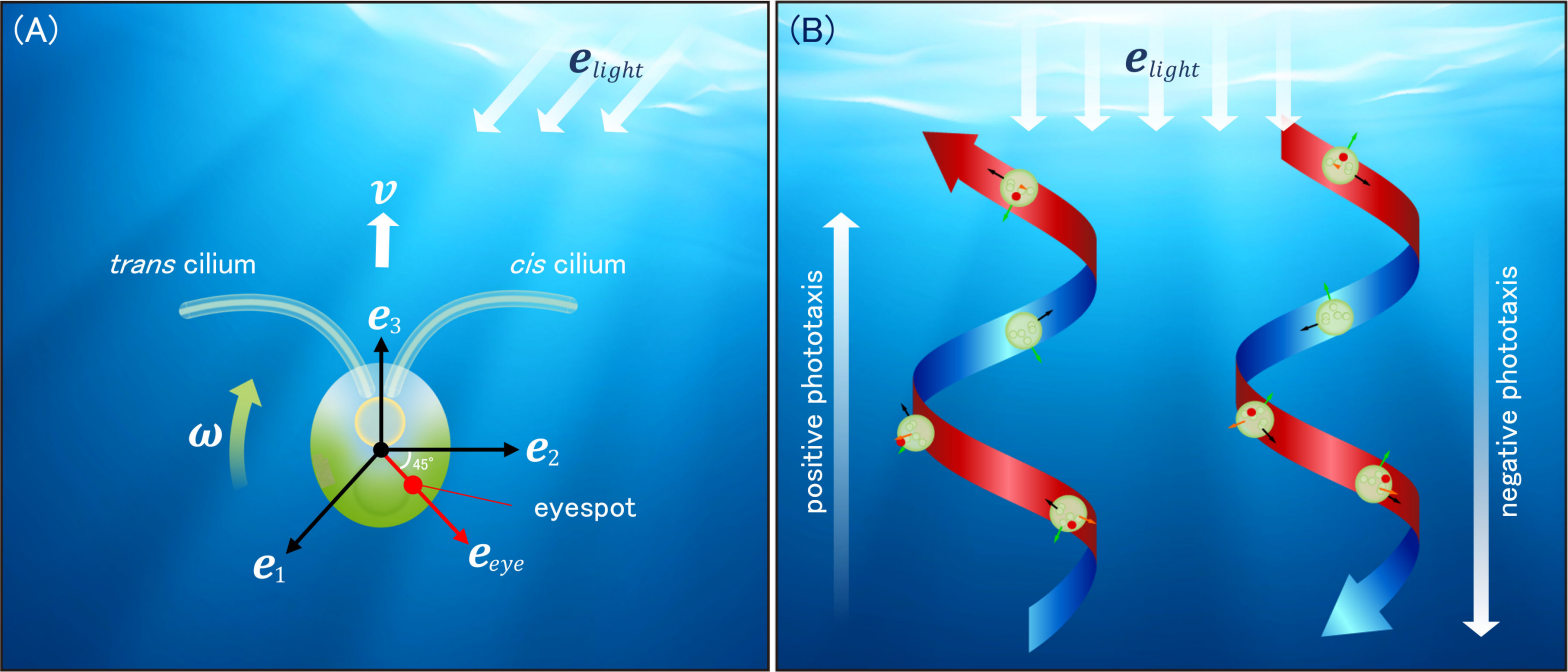
Phototactic movement. (A) Schematics of the body frame $e_{1}-e_{2}-e_{3}$ and directions of eyespot $e_{eye}$ and light $e_{light}$. v and ω are translational and angular velocities, respectively. *Chlamydomonas* has two (*cis* and *trans*) cilia. The strokes of two cilia are three-dimensional, and a torque is generated that rotates the cell counterclockwise around the $e_{3}$ axis. The beating force of the two cilia changes in response to the light stimulus received by the eyespot. This results in the cell rotating around the $e_{1}$ axis, thereby altering the direction of the helical orbit and inducing phototaxis. (B) In two states, the eyespot maintains a constant angle relative to the light source. One state corresponds to a helical orbit towards the light source (left: positive phototaxis), while the other state corresponds to a helical orbit away from the light source (right: negative phototaxis).

Jékely stated in his review paper [26] that in order to gain an understanding of the mechanism of the deterministic phototaxis, it is first necessary to note that the body shape of the microorganisms is (i) stable and (ii) polarized. Property (i) implies that their bodies can be regarded as rigid. Property (ii) allows us to define the three body axes with respect to the body. These are the anterior (A)–posterior (P), dorsal (D)–ventral (V), and, if possible, the left–right axes. Given that cilia responsible for generating propulsive forces are fixed to the body, it is reasonable to express the translational velocity v and angular velocity ω of the microorganism in terms of their body axes as $v=\sum_{i=1}^{3}v_{i}\left(t\right)e_{i}$ and $\omega =\sum_{i=1}^{3}\omega_{i}\left(t\right)e_{i}$, where $e_{i}$ (*i* = 1, 2, 3) represent the unit vectors indicating the directions of body axes, as illustrated in figure 3A. $v_{i}$ and $\omega_{i}$ are the components of translational and angular velocities, respectively. Furthermore, in Stokes flow, the thrust force and torque generated by the cilia are always balanced by hydrodynamic drag force and torque. Consequently, the velocity v and angular velocity ω of the microorganism are determined solely from the ciliary beat at the present time *t*. Assuming the microorganism as a rigid body, its configuration is defined solely by its position r(t) and the directions of its body axes $e_{i}(t)$ [61,62]. The time evolution equations for these variables are

(3.1) ,$$\dot{r}=v$$

(3.2) $${\overset{˙}{e}}_{i}=\omega \times e_{i}\;\;\;\;\;(for\;\;i=1,2,3),$$

where the dot indicates the time derivative of the quantity, and the symbol ‘×’ denotes the cross product of the two vectors. Once the values of $v_{i}$ and $\omega_{i}$ are determined, the movement of the microorganism can be completely determined via equations (3.1) and (3.2).

In 1993, Crenshaw observed and provided an intriguing assertion regarding the trajectory drawn by microorganisms [63]. As previously stated, the trajectory of the microorganism is entirely defined by the values of $v_{i}$ and $\omega_{i}$. In mathematics, on the other hand, the trajectory of an object moving in three-dimensional space can be described by the curvature and torsion of the trajectory, which is known as the Frenet–Serret formulas. Therefore, there must be a correlation between the curvature and torsion of a trajectory and the translational velocity v and angular velocity ω of the swimming microorganism. Crenshaw established a connection between the movement of swimming microorganism and the geometrical problem that determines the curvature and torsion of the trajectory [63]. It is important to note that the description of the trajectory with v and ω provides more information than that with the curvature and torsion, because the former has 6 degrees of freedom, while the latter has 2 degrees of freedom. Since the description with the curvature and torsion is much simpler than that with v and ω, it is a more suitable starting point for investigating the taxis of microorganism [64,65]. Indeed, Friedrich and Julicher, along with other research groups, have described the chemotaxis of sperm cells by assuming a relationship between the curvature and torsion of the trajectory and the chemical stimulus s, which corresponds to the concentration of chemoattractant at the position of the cell [66,67].

### (b) Phototactic movement

In contrast to chemotaxis, the description using v and ω is more useful for phototaxis, as the light is a vector quantity, and the photoreceptor on the organism is directive, i.e. its sensitivity depends on the relative angle between the incident light and the normal direction of the photoreceptor. In order to evaluate the stimulus s, it is necessary to ascertain the directions of body $e_{i}$. Indeed, several studies on the phototaxis of microorganisms, including *Chlamydomonas* [68,69], *Volvox* [70] and *Gonium* [71], have obtained the relations between the light stimulus s and the responses in v and ω from experimental observations. These studies have succeeded in reproducing phototaxis of these microorganisms.

Thus, when examining the taxis of microorganisms in three-dimensional space from a physical perspective, it becomes evident that the most biological and essential aspects of this behaviour is the relationship between the stimulus s(t) that the organism receives and v(t) and ω(t). This formulation can describe various forms of taxis exhibited by different species. The distinction between different types of taxis and species appears in the manner of evaluating the stimulus s and the relationship between s, v and ω. For instance, in the case of chemotaxis, s represents the chemoattractant concentration; whereas, in the context of phototaxis, s denotes the inner product of light direction and the direction of eyespot. The relationship between s, $v_{i}$ and $\omega_{i}$ may be named ‘ethological constitutive equation’ of microorganisms. The ethological constitutive equation is typically represented by a set of ordinary differential equations that incorporate the adaptation to the stimulus [66,69,70].

As an example, consider the case of *Chlamydomonas*, which is one of the most extensively studied microorganisms exhibiting phototaxis. *Chlamydomonas* has two (*cis* and *trans*) cilia at the anterior region and an eyespot on the lateral side. The relative positions of these organelles are fixed, allowing us to define the body axes as illustrated in figure 3A. The direction of the eyespot is given as $e_{eye}=(e_{1}+e_{2})/\sqrt{2}$. The beating of two cilia propels the cell forward, simultaneously rotating about A–P and D–V axes [72]. We can thus express $v=v^{c}e_{3}$ and $\omega =\omega_{1}e_{1}-\omega_{3}^{c}e_{3}$, where $v^{c}$ and $\omega_{3}^{c}$ are treated as positive constants. It should be noted that $\omega_{1}$, the angular velocity about $e_{1}$, can change with time. This is because when the eyespot receives a light stimulus, the two cilia alter the beating patterns, resulting in the generation of angular velocity about $e_{1}$. There are observations that when the light stimulus increases (step-up stimulus) the *trans* cilium beat strongly and the *cis* cilium beat weakly, whereas when the light stimulus decreases (step-down stimulus) the *trans* cilium may beat weakly and the *cis* cilium beat strongly [69,73,74]. This property is expressed as $\omega_{1}=\omega_{1}^{c}-p(t)$, where $\omega_{1}^{c}$ is a constant and p represents the light response of the cilia. The time evolution of p is, for instance, given as


(3.3) $$\overset{˙}{p}=\left(s-h-p\right)/t_{1},\;\;\overset{˙}{h}=\left(s-h\right)/t_{2}.$$

Equation (3.3) is a phenomenological constitutive equation that is derived from the consideration of chemical reactions within the body of microorganisms. The variable p is an observable, while h is a hidden variable describing an intrinsic dynamics responsible for adaptation. $t_{1}$ and $t_{2}$ indicate the rapid response time and the slower adaptation time, respectively. The quantity s is the light stimulus received by the eyespot, which can be expressed as $s\left(t\right)=-I_{0}e_{eye}\cdot e_{light}$, where $e_{light}$ is the direction of incident light and $I_{0}$ is a positive constant. The eyespot is sensitive to the light incident from the outside of the cell but not to the light passing through the cell.

Equation (3.3) provides the relationship between s and $\omega_{1}$ [69], which represents the ethological constitutive equation of *Chlamydomonas*. As the cell rotates around the $e_{3}$ axis, the value of s undergoes a temporal change. As long as s undergoes change, $\omega_{1}$ also changes, resulting in the direction of the cell’s helical orbit continuing to undergo change. However, if there is a state where the angle between $e_{eye}$ and $e_{light}$ remains constant during cell movement, s becomes constant, p becomes zero, and $\omega_{1}$ remains constant, i.e. $\omega_{1}=\omega_{1}^{c}$. In this state, phototaxis is fully achieved. In mathematical terms, the aforementioned state corresponds to the steady solution of equations (3.1)–(3.3).

In the case of *Volvox*, many somatic cells with eyespots are distributed on the surface of a spherical colony. When activated in the presence of light, the somatic cells demobilize their flagella, allowing the *Volvox* to rotate and swim toward the light source. Even in this case, the response of each somatic cell can be expressed by equation (3.3) to describe the phototaxis of the entire colony [70]. When the axis of symmetry of the *Volvox* colony becomes parallel to the direction of light, the angle between $e_{eye}$ and $e_{light}$ of each somatic cell remains constant, and $\omega_{1}$ remains constant. In this state, phototaxis is fully achieved for the *Volvox*. Equation (3.3) is an ethological constitutive equation that has been widely used to describe microbial taxis, including chemotaxis in bacteria and sperm [66,75].

### (c) Positive and negative phototaxis

Although equations (3.1) and (3.2) are concise, the equations with the ethological constitutive equation provide insight into the phototaxis of microorganisms, such as the switching of phototactic sign [76], whose mechanism is still an open question in the field of phototaxis. In fact, the steady solution of equation (3.1)–(3.3) is twofold. One is the helical orbit moving towards the light source (positive phototaxis), and the other is the helical orbit away from the light source (negative phototaxis), as illustrated in figure 3B. The state that is ultimately selected is contingent upon the ethological constitutive equation between the light stimulus s received by the organism and the response of the organism expressed by the change in v and ω.

Jékely and Foster proposed that if the response of the ciliary beating is delayed with respect to the light stimulus, the phototactic sign can change [26]. This hypothesis was corroborated by mathematical models [77]. Equations (3.1) and (3.2) also demonstrate the assertion made by Jennings in 1899 [78], namely that the same side of the cell body is always directed toward the outside of the spiral trajectory [63]. The equations (3.1) and (3.2) exhibit that the sign of $\omega_{1}^{c}$ determines the relative position of eyespot of *Chlamydomonas* with respect to the helical orbit. When $\omega_{1}^{c}>0$, the eyespot is oriented outward, while when $\omega_{1}^{c}<0$, it is oriented inward. Furthermore, the relative direction of the eyespot does not relate to the sign of phototaxis [74,77]. The value of $\omega_{1}^{c}$ can be estimated from the trajectory of the cell, which comprises the pitch, radius of the helical trajectory and swimming velocity.

## 4. Responses to surfaces

Microorganisms are often found in areas bounded by walls or water surfaces. For instance, biofilms, which are constructed by the accumulation of bacteria, frequently develop on solid walls and water surfaces. Such biofilms have been involved in health problems such as tooth decay and catheter contamination. Microorganisms that feed on biofilms need to remain on the wall surface in order to feed for a prolonged period of time. What forces enable these microorganisms to remain on the wall surface? The mechanical conditions of a solid wall and a water surface are significantly different, because the velocity slips at the water surface but the velocity is zero at the solid wall. Which is more challenging for microorganisms: to remain at the water surface or at the solid wall? Moreover, in the case of microbial symbionts, the symbionts undergo complex internal migrations to reach specific cellular compartments or housing organs [28]. The physical environments in which the symbionts reside are subject to significant geometric constraints. The question thus arises as to how such migration is achieved. In order to address these questions, it is necessary to gain an understanding of the behaviour of microorganisms in the vicinity of interfaces. Thigmotaxis is a behavioural response to physical contact with surfaces. This section will discuss the behaviour of microorganisms on walls and water surfaces from a physical perspective.

When microorganisms are far away from a wall boundary, the effect of the wall on microbial swimming is small, and it is difficult for them to recognize the wall. This is because the velocity at which the microorganism is affected by the wall typically decays with the square of the distance between the microorganism and the wall, and the angular velocity decays with the cube of the distance from the wall [79]. Conversely, in situations where the microorganism is in close proximity to the wall, fluid forces, steric repulsive forces and adhesive forces act on the microorganism. Fluid forces result from the pressure and friction forces between the microorganism and the wall and play an important role in the stable swimming of bacteria in the vicinity of the wall surface [80]. When the distance between the microorganisms and the wall surface is small, lubrication flow occurs within the narrow gap, and lubrication forces are exerted [81]. The lubrication force can be considerable in magnitude, exerting a profound influence on the swimming behaviour of microorganisms. It can act to prevent them from remaining near the wall or making it more difficult for them to leave the wall. Adhesion forces are attractive forces that draw microorganisms to the wall, while steric repulsive forces act to repel them away from the wall. These forces are of significant importance during the initial stages of biofilm formation, and have been extensively studied [82]. In the following sections, we present recent advances in our understanding of microbial behaviours near interfaces, focusing on bacteria, microalgae and ciliates as examples.

### (a) Behaviours of bacteria

The behaviour of bacteria near a solid wall has been extensively studied using *E. coli*. Berke *et al*. [83] investigated the distribution of *E. coli* between two glass plates and observed a significant increase in the concentration of cells near the walls. The mechanism by which *E. coli* accumulate near the walls is explained by their hydrodynamic interaction with the wall and their tendency to swim parallel to the wall after collision. Bacterial accumulation on walls has been observed in the gut of zebrafish [84], suggesting that this phenomenon may be widespread in nature.

In the vicinity of a wall, *E. coli* swim stably with a circular trajectory. As *E. coli* swim by rotating helical flagella, the flagella and the body rotate in opposite directions, such that the sum of the torques is zero. This results in opposing lubrication forces acting on the flagella and the body, which is the mechanism that generates the circular trajectory. The mechanism of stability near a wall is elucidated by hydrodynamic interactions with the wall [80,85]. We note that when *E. coli* swim near a free surface (liquid–air interface), they swim stably with a circular trajectory in the opposite direction to that observed near a wall [86]. This is because the sign of the surface-induced forces is reversed when the rigid wall is replaced by a free surface.

In recent years, the behaviour of bacteria under very confined conditions has been reported. Tokárová *et al*. [87] examined the behaviour of five bacterial species (*Vibrio natriegens*, *Magnetococcus marinus*, *Pseudomonas putida*, *Vibrio fischeri* and *E. coli*) in microchannels presenting varying degrees of confinement and geometric complexity. In the moderate confinement, both hydrodynamics and steric interactions play a pivotal role. These factors either align the swimming direction of bacteria within the channel, or disrupt the swimming direction, thereby restricting their migration. In strong confining environments, however, the motility is mainly governed by the steric interactions between the bacteria and the surrounding walls. Bhattacharjee & Datta [88] observed that the run-and-tumble behaviour of *E. coli* is altered to hopping-and-trapping behaviour in three-dimensional porous media. Furthermore, porous environments also affect bacterial behaviours such as chemotaxis. Bhattacharjee *et al.* [89] demonstrated that chemotactic migration of *E. coli* in porous media is achieved by modulating the degree of reorientation to bias their hopping orientation, resulting in more hops along the concentration gradient.

Lynch *et al*. [90] investigated the symbiotic process between marine bacterium *Vibrio fischeri* and Hawaiian bobtail squid, *Euprymna scolopes*. Instead of performing the real *in vivo* experiments, they employed microfluidics and capillaries to construct a diorama environment representing the symbiotic process. As Ishikawa [91] notes, diorama environments are well-controlled miniature models of realistic environments. Lynch *et al*. demonstrated that *V. fischeri* cells are able to efficiently escape from the narrow channel by reducing sharp reversals in trajectory. They also attempted an experiment in which two contradictory stimuli were presented simultaneously: the tendency to escape from the narrow channel and the tendency to move towards a chemoattractant. Even in the presence of the chemoattractant, the cells avoided the narrow channel, which illustrates the enormous influence of wall surfaces on the behaviour of bacteria.

### (b) Behaviours of microalgae

The behaviour of microalgae *Chlamydomonas reinhardtii* near a solid wall was investigated by Kantsler *et al*. [92]. They measured the incidence and scattering angles during the interactions with the wall, and found that the scattering angles do not correlate with the incidence angle. This is due to the fact that the cilia make multiple contacts with the surface. They concluded that the surface scattering of *C. reinhardtii* is governed by ciliary contact interactions rather than long-range hydrodynamic interactions.

The microalgae *Volvox* exhibits gravitaxis due to its bottom-heaviness and basically swims vertically upward. Drescher *et al*. [37] used *Volvox carteri* to examine its behaviours near wall boundaries. In the presence of an upper wall, *V. carteri* that reach the upper wall hover stably just below the wall. In this case, the *V. carteri* acts like a pump, pushing liquid downwards and sucking liquid in from the side. Attracted by this flow, the surrounding *V. carteri* form clusters and move as if dancing a waltz. As the *V. carteri* grow older, however, their sedimentation speed exceeds their swimming speed, and they sink towards the bottom wall. Such *V. carteri* do not collide with the bottom wall, but hover slightly above it. *V. carteri* near the bottom wall also create a mutually attracting flow and dance a minuet [37]. These behaviours of *V. carteri* near the walls have been well reproduced by numerical simulations, and the mechanism can be well explained by hydrodynamics [93].

Noselli *et al*. [94] investigated the effect of strongly confined geometry on the behaviour of *Euglena gracilis*. By observing cells swimming in tapered capillaries, they found that the cells can change their mode of locomotion from standard flagellar swimming to fast crawling. This new mode of locomotion was able to accommodate extreme geometric confinement, converting both frictional and hydraulic resistance to propulsion. As the switch in the locomotion mode is triggered by confinement, it is evident that the wall boundary is a significant factor.

### (c) Behaviours of ciliates

The behaviour of ciliates in the vicinity of walls has long been investigated. Jennings [95] observed that a ciliate, *Paramecium*, exhibited a physiological response of escaping from a wall when it bumped against it. The cells escape from the wall by swimming backward, rotating around their posterior end, and then resuming normal forward swimming, which he termed the ‘avoiding reaction’. The avoiding reaction in *Chlamydomonas reinhardtii* is triggered by activation of transient receptor potential (TRP) channels located in the proximal region of cilia [96,97].

**Figure 4.**
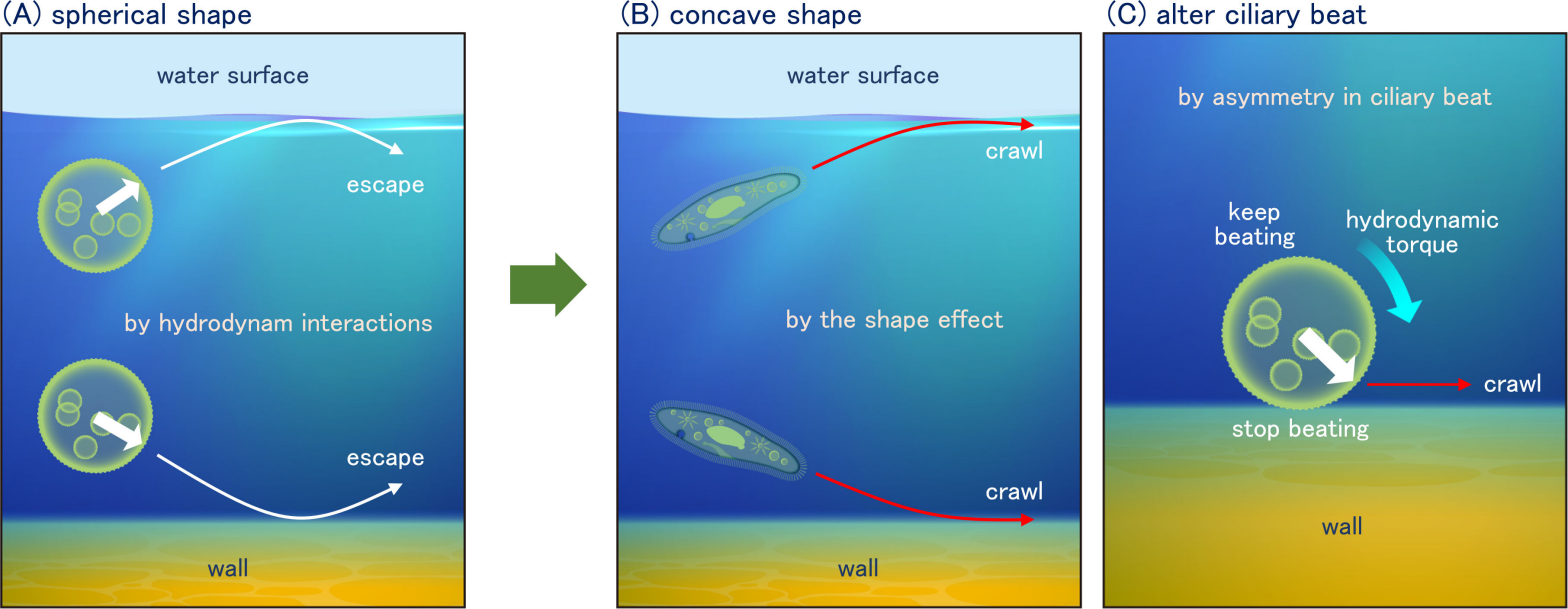
Physical mechanism of behaviours at interfaces. (A) A spherical ciliate is able to escape from interfaces by hydrodynamic interactions. (B) A ciliate with the concave shape can crawl at the interfaces through hydrodynamic and steric forces. (C) A ciliate can remain and crawl on a solid wall by halting the ciliary beat only in the vicinity of the wall.

Ciliates exhibit distinct behaviours at the water surface. Ferracci *et al*. [19] reported the entrapment of ciliates *Tetrahymena thermophile* at the water surface. The swimming speed of the cells was found to be significantly reduced close to the water surface. They explored the influence of chemotaxis and gravitaxis on the surface entrapment, but concluded that the primary cause of the entrapment is hydrodynamics. Manabe *et al*. [29] further investigated the entrapment phenomenon through numerical simulation. They found that body shape has a significant effect on the entrapment phenomenon and the mechanism of entrapment can be explained by the balance between hydrodynamic escaping torque and steric attractive torque (cf. Figure 4B). Okuyama *et al*. [98] used a different *Tetrahymena* strain, i.e. *T. pyriformis*, and observed similar accumulation of cells near the water surface. The entrapment at the water surface appeared to be a property shared by *T. thermophile* and *T. pyriformis*.

Entrapment of ciliates can also be seen on a solid wall. Ohmura *et al*. [18] reported that *T. pyriformis* can remain on the wall, though hydrodynamic torque causes cells to swim away from the wall, as illustrated in figure 4A. They found that *T. pyriformis* senses the collision with the wall and reduces the movement of only the cilia located in close proximity to the wall. This results in an asymmetric force produced by the cilia and a torque that directs the cell towards the wall, as illustrated in figure 4C. This is the mechanism by which *T. pyriformis* is able to remain on the wall surface. Similar crawling motion has also been observed in *Paramecium* [99]. Furthermore, Ohmura *et al*. [14] reported that the kinesthetic sensing of cilia can induce near-wall rheotaxis in *T. pyriformis*.

By contrast, *T. thermophile* cells tend to escape from the solid wall [19]. The difference in the behaviour of *T. thermophile* and *T. pyriformis* at the wall may be attributed to the disparate response of their cilia to the wall. *T. pyriformis* ceases the beating of cilia that attach to the wall, whereas *T. thermophile* does not. Ishikawa & Kikuchi [100] examined the behaviour of *T. thermophile* between two flat plates with an acute angle. This setup yields the formation of a *dead end* line where the two plates come into contact. They demonstrated that cells can hydrodynamically escape from the dead end line using a lubrication torque. They also showed that some cells stuck between the two plates exhibited the avoiding reaction, indicating that *T. thermophile* can escape from the dead end through both hydrodynamical and biological responses.

Kunita *et al*. [101] reported that *Tetrahymena* can even learn the geometric constraints of their surrounding environments. They observed that cells briefly trapped in a small water droplet exhibited a tendency to reproduce circular swimming trajectories when released. The diameter of the circular trajectories and their duration reflected the size of the droplet and the duration of confinement. Echigoya *et al*. [102] examined the behaviour of a ciliate *Stentor coeruleus* in confined geometries. Interestingly, the cell tends to adhere in the narrow crescent area, which suggests that the cells are capable of recognizing the shape of the surrounding space and modifying their behaviour in accordance with this recognition. These findings demonstrate that ciliates are not only capable of responding to surfaces instantaneously, but also of memorizing their environment and exhibiting long-term responses.

## 5. Responses to flow

In the 1990s, the prevailing assumption in oceanography was that the distribution of marine microorganisms was roughly random and homogeneous [103]. However, over the past decade, it has become evident that spatial gradients of various physical quantities, such as velocity and viscosity, result in heterogeneous distributions [104]. This is the consequence of the interaction between cell motility and the mechanical environment at the cellular scale, demonstrating how mechanically induced taxis shapes the macroscopic microbial distribution. This section presents a review of research on the emergence mechanisms of rheotaxis, viscotaxis and turbulent-induced heterogeneous cell distribution, with a particular focus on the interaction between flow field and cell motility.

### (a) Mechanism of rheotaxis

Rheotaxis is defined as directed movement in response to an external fluid flow. It has been observed in unicellular swimming microorganisms such as *Escherichia coli* [105], *Bacillus subtilis* [106], *Tetrahymena pyriformis* [14], *Chlamydomonas reinhardtii* [107] and mammalian spermatozoa [30,31,108,109]. It is anticipated that positive rheotaxis, the capacity to orientate and swim against the fluid flow, will serve as a guidance mechanism for mammalian sperm swimming in the female reproductive tract [30].

Since the discovery of the sperm rheotaxis in 1872 [110], there has been a debate as to whether the mechanism is due to an active biological response or a physical passive response. For instance, Miki & Clapham [30] observed a positive rheotaxis of mouse spermatozoa *in vivo* and proposed that Ca^2+^ influx via CatSper and associated flagellar waveform change are essential for the development of positive rheotaxis. However, no Ca^2+^ influx into the flagellum was observed during the human sperm rheotaxis-turn [109], suggesting that there is no active signal transduction during human sperm rheotaxis. The acquisition of rheotaxis in swimming microorganisms is now considered to result from passive physical phenomena rather than an active biological response by mechanosensing. A number of mathematical models have been developed to clarify the physical mechanisms of rheotaxis [14,31,111,112].

**Figure 5.**
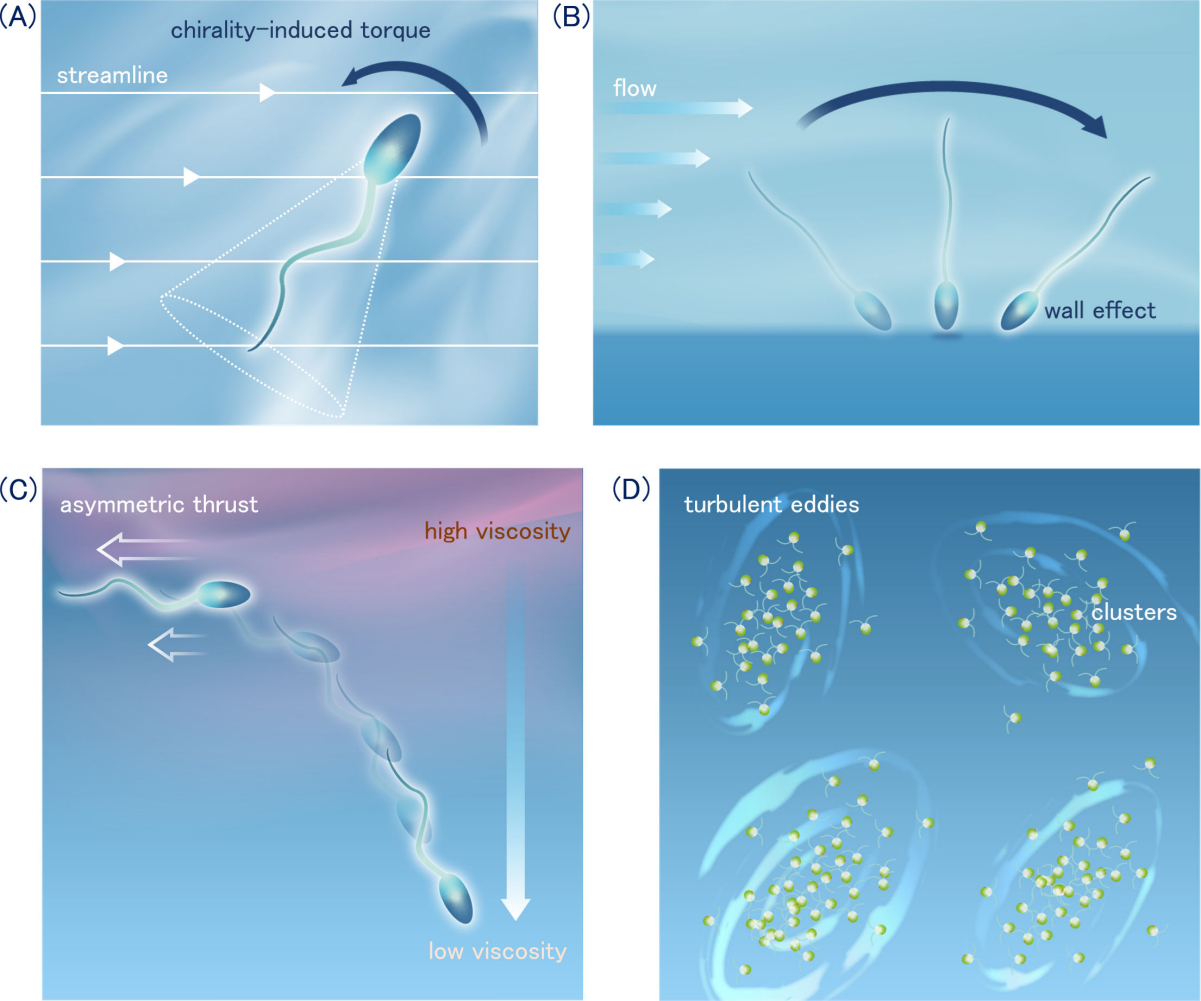
Response to flow. Two mechanical models of positive rheotaxis, (A) chirality and conical waveform of sperm flagellum causes a torque that directs the cell perpendicular to the shear plane, and (B) wall-induced weathervane model: rotational torque generated by velocity gradients as the microorganisms move towards the wall. (C) Negative viscotaxis. Swimming towards the low viscosity region is caused by asymmetric thrust force according to the viscosity gradient. (D) Turbulent eddies can form heterogeneous patches of microorganisms due to the interaction between cell motility and fluid flow.

Two principal physical factors by which cells exhibit rheotaxis are the chirality effect and the wall effect under velocity gradient. The chirality model is based on the conical or helical waveform of sperm flagellum. The posterior part of the flagellum experiences a greater hydrodynamic force than the anterior part, resulting in the generation of a torque that reorients cells perpendicular to the shear plane (figure 5A) [109,111,113]. This torque is not contingent on the presence of a wall boundary, indicating that the rheotaxis is not constrained to liquid–solid interfaces, as illustrated in figure 5A. In addition, the chirality effect can also generate drift of flagellated bacteria relative to the bulk flow [106].

The size of swimming microorganisms is so small that a linear approximation to the velocity gradient of fluid flow is generally applicable. In a simple shear flow, a constant angular velocity is generated, which exerts fluid torques that act to rotate the cell body. When a cell swims in close proximity to a wall, it directs upstream analogous to a weathervane, as illustrated in figure 5B [31,105,112,114]. The stability of the upstream swimming has been attributed to the hydrodynamic and steric repulsive forces exerted by the wall. The effects of walls have been demonstrated to induce positive rheotaxis even in two-dimensional planar sperm flagellar waveforms [112] and in ciliated swimming microorganisms [14]. These wall effects may assist in the establishment of a robust habitat near the liquid–solid interface and in the long-range guidance mechanisms of mammalian sperm in the female reproductive tract.

### (b) Response to viscosity gradient

There is also an interesting physical tactic behaviour, viscotaxis, in response to spatial gradients in viscosity. Viscotaxis has mainly been observed in bacteria, *Spiroplasma* and *Leptospira*, which exhibit positive viscotaxis [115,116] and move up viscosity gradients, whereas *E. coli* exhibits negative viscotaxis [117,118]. As bacteria often swim in heterogeneous viscous environments, such as biofilms and mucus layers, the acquisition of viscotaxis is thought to provide a survival advantage by remaining in a viscous environment favourable to their habitat. Research on the underlying mechanisms of viscotaxis does not have as long a history as rheotaxis, but theoretical locomotion analyses of microswimmers in viscosity gradient environments have been performed in recent years [119–122].

Liebchen *et al*. [121] investigated the minimal physical design of viscotactic swimmers, consisting of three linked beads arranged in a triangle, with prescribed propulsion forces in slightly varying viscosity fields. They showed that the viscous drag acting on body parts at high viscosity dominates the drag acting on body parts at low viscosity. The asymmetric drag induces a torque on the swimmer to turn up the viscosity gradients. Datt & Elfring [119] theoretically investigated the dynamics of active particles in a linear viscosity gradient field using a spherical squirmer model with prescribed surface velocities. They derived that, under small viscosity gradient conditions, the angular velocity ω generated by the viscosity gradient is $\omega =-\frac{1}{2\mu_{0}}v\times \nabla \mu$, where v is the translational velocity, $\mu_{0}$ is the mean viscosity and ∇μ is the viscosity gradient. The squirmer exhibited negative viscotaxis regardless of the swimming mode and was shown to be oriented towards the low viscosity side unless the gradient was perfectly aligned.

The dynamics of microorganisms in a non-uniform viscous environment not only alter microbial movement, but also affect nutrient influx [120]. Non-uniform viscous environments alter the local diffusivity of nutrients, suggesting that the relationship between the direction of microbial movement and the direction of the viscosity gradient can alter nutrient uptake by microorganism. In addition, in environments such as the ocean, there is not only a viscosity gradient but also a density gradient of the fluid. Densitaxis caused by the density gradient of the surrounding fluid has recently been reported by Shaik & Elfring [123]. Although the phenomenon has not been experimentally confirmed, taxis of microorganisms may also be caused by a density gradient.

### (c) Response to turbulence

Most flows observed in nature are turbulent flows, where inertia is dominant. By contrast, in the flows to which microorganisms are exposed, the effect of inertia is negligible due to the very small size of the bodies, and the viscous effect becomes dominant. For this reason, the impact of turbulence on microbial ethology has rarely been discussed. However, the importance of its effects on motile microorganisms has recently been reported [56,104,124–127].

For example, stirring and mixing effects of turbulence on nutrient patches have been shown to favour motile bacteria over non-motile competitors in terms of fitness [104,126,127]. Bacteria in the ocean should experience turbulence as a smooth, slowly changing velocity gradient, as the Kolmogorov scale, i.e. the minimum turbulent eddy scale, is estimated to be around 1–10 mm [127]. Turbulent mixing produces variance in the distribution of dissolved nutrients on scales as small as the Batchelor scale; 30 to 300 µm [104,126,127]. This scale is about 100 times the length of a bacterial body, but is large enough to be covered in a few seconds by motile bacteria with chemotactic velocities and exploit during the lifetime of a typical nutrient patch [104,127]. Non-motile bacteria, on the other hand, can only utilize diffusive fluxes of nutrients at the cellular scale. There is a trade-off between the energetic cost of locomotion in eddies and the benefit of nutrient absorption, and numerical calculations indicate the existence of an optimal swimming speed for swimming in turbulent flow [127].

Turbulent eddies have also been reported to affect microbial accumulation [56,125]. In high vorticity regions in turbulent flows, phytoplankton *Chlamydomonas augustae* were found to cluster more stably than cluster formation by gyrotaxis [56]. These cell clusters were not observed in dead cells and are thought to be formed by the interaction between turbulent eddies and cell motility. Cell motility can prevail over turbulent dispersion to create strong fractal patchiness, with local phytoplankton concentrations increasing more than 10-fold [125]. Turbulence in environmental flows can promote the formation of patchiness by motile phytoplankton and control their ecological distribution.

## 6. Summary and prospects

In this review paper, we have discussed the physical mechanisms of gravitaxis, phototaxis, thigmotaxis and rheotaxis. In all of these phenomena, microbial behaviour can ultimately be attributed to the physical stimuli to which the microorganisms are exposed and their responses, which manifest themselves as behaviours. In §3, the relationship between physical stimuli and microbial behaviour is described as a relational equation between the stimulus s, the translational velocity v and the angular velocity ω of a microorganism, and this equation is termed the ‘ethological constitutive equation’. In future research, it will be valuable to discover the ethological constitutive equations for a wide variety of microorganisms. It would also be interesting to classify them into a number of cohesive groups and identify general trends. The knowledge would lead to the fundamental ethological algorithms of microorganisms.

This paper assumes that the microorganism is exposed to only one type of stimulus. However, in real outdoor environments, it is common for multiple stimuli to act simultaneously. The ethological constitutive equations for multiple stimuli acting simultaneously, such as when light, flow and chemical stimuli are present, are unclear. Microorganisms may be subjected to both attractant and repellent stimuli at the same time, and in such cases it is suspected that the superposition of responses to individual stimuli may not be adequate to describe them. In order to derive the ethological constitutive equations in such complex environments, attempts have been made to construct experimental setups that simplify the complexity of outdoor environments while retaining essential complexity. Experimental systems built for this purpose are called diorama environments [91,128,129]. These emerging methodologies will lead to new developments in the physical study of microbial taxis.

The above discussion on the physical features of taxis does not exclude the involvement of physiological responses in taxis. The degree of control of physical and physiological phenomena differs among types of taxis. Phototaxis in *Chlamydomonas* is triggered by photoreception in the eyespot and the swimming is steered by controlling the balance of the forces generated by two cilia [130,131]. Gravitaxis may mostly be explained by the shape asymmetry model, but some *Chlamydomonas* mutants with intact shape but defective membrane excitability are defective in gravitaxis [132]. *Tetrahymena* crawl on the wall by stopping the motility of cilia close to the wall presumably through mechanosensation [18]. Therefore, how stimulus perception is linked to motility control is another important aspect of the study on taxis.

The behaviour of microorganisms as a population is often crucial in various fields, including industry, fisheries and medicine. If the suspension of microorganisms is sufficiently dilute, there may be no significant differences between the behaviour of an individual microorganism and that of a group. In dense suspensions, however, spontaneous flow may occur, altering the physical stimuli and leading to different behaviours [133]. Thus, the ethological constitutive equation for a population of microorganisms also needs to be discovered. When discussing the behaviour of microorganisms at the macro scale, it may be more appropriate to describe the number density of microorganisms as a continuum, rather than describing the behaviour of individual microorganisms, as the scale of the phenomenon is sufficiently larger than the scale of the microorganism. In such cases, the ethological constitutive equation would not relate the stimulus s to the velocities v and ω, but would need to relate the stimulus s to the particle stress tensor, drift velocity and the diffusion tensor appearing in the governing equations of the continuum models [133]. We expect that the accumulated knowledge of the ethological constitutive equations will lead to a better understanding of the biological, medical and engineering phenomena produced by the behaviour of microorganisms.

## Data Availability

This article has no additional data.
